# Extrusion Behavior, Osmolality and pH of a Reconstituted Conditioned-Medium Cosmetic Product: A Pilot Physicochemical Comparison of Two Reconstitution Vehicles

**DOI:** 10.3390/molecules31214033

**Published:** 2026-11-09

**Authors:** Miho Nemoto, Hidekazu Yamada

**Affiliations:** 1 Nemoto Dermatology, Plastic Surgery and Cosmetic Dermatology Clinic, 4-8 Takamatsu-cho, Nishinomiya 663-8204, Hyogo, Japan; 2 Anti-Aging Center, Kindai University, 3-4-1 Kowakae, Higashi-Osaka, Osaka 577-8502, Japan; 3 Department of Dermatology, Faculty of Medicine, Kindai University, 1-14-1 Mihara-dai, Minami-ku, Sakai, Osaka 590-0197, Japan; 4 Department of Dermatology, Nara Hospital, Faculty of Medicine, Kindai University, 1248-1 Otoda-cho, Ikoma, Nara 630-0293, Japan

**Keywords:** conditioned medium, cosmetic formulation, reconstitution vehicle, extrusion behavior, osmolality, pH, sodium hyaluronate, preliminary study

## Abstract

Lyophilized cosmetic biologics are supplied as an active powder and a base solution, but the physicochemical properties of the reconstituted preparation are seldom reported. In this preliminary study one production lot of a commercial human stem cell conditioned medium extract was reconstituted either with the supplied base solution or with 0.9% sodium chloride, at the same active concentration, and compared in a standardized extrusion test (26 G orifice, 50 g load, 0.2 mL, 25 °C). Replicates were repeated measurements on one preparation of each fluid rather than on independent preparations, so the data are summarized descriptively. The saline-reconstituted preparation gave a median extrusion time of 3.05 s (range 2.52–3.58) and the manufacturer-directed preparation 39.63 s (20.85–107.97), a 13-fold difference; the latter could not be aspirated into a freezing-point-depression osmometer. The base solution alone did not extrude within 60 s at 50 g, but did at 100 g, with medians of 8.03, 5.98 and 6.48 s in three independently opened vials. Doubling the load changed the observed extrusion time of the reference saline by a factor of 2.5 and that of the base solution by at least nine, which suggests a dependence of flow on the applied stress that a Newtonian fluid would not show, although the two loads were measured in separate sessions. The saline-reconstituted preparation measured 374 mOsm/kg against 286 mOsm/kg for its diluent; both preparations had a pH of 5.0 and both diluents 5.5, so the diluent did not determine the final pH. These preliminary observations require confirmation with independently prepared replicates and indicate that such properties are worth measuring on the final reconstituted fluid rather than inferred from the ingredient declaration.

## 1. Introduction

Cosmetic formulations built around biologically derived macromolecules—conditioned media, extracellular vesicles (EVs), growth factors, and signaling proteins—face a constraint that small-molecule cosmetic actives do not. The stratum corneum restricts passive percutaneous absorption to compounds of roughly 500 Da or less [1], whereas EVs are lipid-bilayer-delimited particles typically in the 30–200 nm range [2,3] and Klotho is a large transmembrane/secreted protein [4,5]. Passive topical application of such ingredients therefore relies on effects at or near the skin surface, or on prolonged application; a recent 12-week pilot study of a Klotho-containing serum reported investigator-assessed improvements in photodamage, fine lines and dyschromia under a twice-daily regimen [6].

Energy-based devices provide an alternative route. Fractional laser- and microneedle-assisted delivery create transient physical conduits through the barrier, and the pharmacokinetic consequences of this approach have been characterized in some detail for small molecules [7,8]. Microneedle radiofrequency is attractive in this context because a single device performs two functions: insulated micro-electrodes deposit thermal energy in the reticular dermis to produce coagulation zones and subsequent remodeling, while the needle tracks themselves constitute micro-channels [9].

Attention in this field has centered on the active ingredient and device parameters. The vehicle has been largely overlooked, yet it presents two distinct problems.

The first is thermal. The intradermal temperatures required for collagen contraction are well above the range in which EV preparations retain their physical and functional characteristics. The EV literature is explicit that vesicle integrity is sensitive to thermal handling: storage temperature alters vesicle size distribution, particle recovery, and downstream function [10,11,12], stabilization requires deliberate control of buffer and temperature [13], lyophilization is adopted specifically to avoid losses in the liquid state [14], and the composition of the suspending medium is a determinant of preparation stability [15,16]. This literature is cited here only to motivate attention to the vehicle. No thermal measurement, and no measurement of vesicle integrity, was made in the present study, and the thermal problem is not addressed further below.

The second concerns the physicochemical properties of the reconstituted fluid itself. Lyophilized actives of this class are supplied as two vials—an active powder and a cosmetic base solution. Such base solutions commonly contain thickening polymers, most often sodium hyaluronate, because retention on the skin surface requires resistance to flow, whereas delivery into conduits of finite diameter requires the opposite. Furthermore, once a barrier-breaching device is used, the osmolality and pH of the applied fluid become relevant in a way that they are not for application to intact skin. None of these properties is stated on the packaging of such products, and all are routinely assumed rather than measured.

We measured them. This report compares the extrusion behavior, osmolality, specific gravity, and pH of two reconstituted preparations of one such product. It is a preliminary, hypothesis-generating study of a single production lot, conducted with repeated measurements on single reconstituted samples rather than with independently prepared replicates; its purpose is to establish whether differences large enough to be worth investigating exist, not to characterize the product or the class.

## 2. Materials and Methods

### 2.1. Test Material and Preparations Compared

The active material was a lyophilized human stem cell conditioned medium extract marketed for cosmetic use (KLOSOME^®^, Elastem Co., Ltd., Hwaseong-si, Gyeonggi-do, Republic of Korea; International Nomenclature of Cosmetic Ingredients (INCI) name: Human Renal Stem Cell Conditioned Media Extract), commercially described as containing Klotho and extracellular-vesicle-related components. Neither component was verified in the present study (Section 4.3). The product is manufactured by Elastem Co., Ltd. (Hwaseong-si, Gyeonggi-do, Republic of Korea) and distributed by EHL Bio Co., Ltd. (Uiwang-si, Gyeonggi-do, Republic of Korea). The units examined were those placed on the Japanese market and were obtained from Lekarka Co., Ltd. (Tokyo, Japan) on the authors’ request; they were requested on a paid basis but no payment has been made, and they are accordingly reported as having been supplied without charge. The circumstances are set out in the Conflicts of Interest statement. The Japanese-language label applied to the retail carton for the Japanese market was not on the carton at the time of the measurements; a labeled carton was obtained separately from the same company afterwards. The label names INPLUG Co., Ltd. (Osaka, Japan) as the marketing authorization holder and Gerok Co., Ltd. as the distributor, and states that the product is manufactured in Korea and is sold in Japan as a cosmetic. The ingredient list printed on it names the same components in the same order as the specifications reproduced in Table 1. This confirms that we have transcribed the specification correctly; it does not independently verify the composition of the units examined, both documents originating with the manufacturer and the label having been obtained after the measurements rather than with the units examined. It is supplied as a two-vial system: vial A containing 20 mg of lyophilized active powder and vial B containing 5 mL of a cosmetic base solution designated Care Solution. The manufacturer’s directions specify the reconstitution of vial A with the full volume of vial B for topical application. Both vials were stored refrigerated at 4 °C until use. Both carry the same lot marking, 26B01, with an expiry of 4 August 2028, and the same kit lot was used in both measurement sessions.

The quantitative composition of both vials was obtained from the manufacturer’s ingredient specifications (Table 1).

Three features of this composition are relevant to the following discussion. First, sodium hyaluronate at 0.750% *w*/*w* (7.5 mg/mL) is the only macromolecule quantified anywhere in the two specifications; every other individually declared solute is a small molecule present at 0.8% *w*/*w* or below. The undisclosed extract that constitutes 99.3% of vial A will contain macromolecules of its own, which is part of the difficulty set out in Section 4.2; what can be said is that no macromolecule other than the hyaluronate is declared at a stated quantity. Second, vial B contains no emulsifier or film-forming agent; apart from the hyaluronate, it is essentially a salt solution. Third, the sodium chloride content of vial B is 0.800% *w*/*w*, which is close to the 0.9% of physiological saline; therefore, substituting saline for vial B only marginally alters the ionic strength of the vehicle. The final 0.500% is declared in the specification as a premix, but its individual quantities are resolved within it: water 0.300%, butylene glycol 0.150%, and 17 free amino acids totaling 0.050% *w*/*w*. The quantitative composition of vial B is therefore complete, and the osmolality expected of it can be calculated (Section 2.3).

The active constituent of vial A is declared under the single INCI designation “Human Renal Stem Cell Conditioned Media Extract” and accounts for 99.3% of the powder. Its composition is not disclosed.

Two preparations were compared.

- A + B—vial A reconstituted with the full 5 mL of vial B, as directed by the manufacturer.
- A + saline—vial A reconstituted with 5.0 mL of sterile 0.9% *w*/*v* sodium chloride injection, matching the volume of vial B.

The same volume of diluent, 5.0 mL, was added to the powder in both preparations; neither was afterwards made up to a fixed final volume. Each preparation therefore contained 20 mg of powder in 5.0 mL of added diluent, so the concentration of the active was identical in the two and they differed only in the identity of the diluent. Neither volume was verified gravimetrically. Given the composition in Table 1, the substitution removed sodium hyaluronate and 1,2-hexanediol while leaving the sodium chloride concentration essentially unchanged (0.800% versus 0.9% *w*/*v*). Both were prepared immediately before measurement (ex tempore), with the first measurement beginning within 5 min of reconstitution.

### 2.2. Extrusion Test

Rotational rheometry was not available within the timeframe of this study. A comparative extrusion test approximating the geometry of device-assisted delivery was therefore devised using components available in clinical practice.

Each fluid was drawn into a 1 mL tuberculin syringe (Terumo Corporation, Tokyo, Japan; polyethylene terephthalate barrel, slip tip) through an 18 G needle, which was then exchanged for a 26 G × 13 mm needle of 0.45 mm outer diameter and approximately 0.26 mm internal diameter (NIPRO Corporation, Osaka, Japan) before the measurement, with care taken to exclude air bubbles. Loading through the wider bore means that the sample passes through the 26 G orifice only once per replicate, in the extrusion that is timed. The same syringe and needle types were used throughout both measurement sessions described below. All measurements in both sessions were made with syringes from a single carton (Terumo product code SS-01T, lot 251018F, expiry September 2030) and needles from a single pack (NIPRO 26 G × 13 mm, lot 25K303, expiry October 2030), so neither the syringe nor the needle lot varies within or between the two sessions. The sterile water for injection and the 0.9% *w*/*v* sodium chloride injection were taken from the clinic’s routine stock, which is consumed in quantity in daily practice; their lot numbers were not recorded and we do not reconstruct them. The syringe was fixed vertically, and a constant 50 g load was applied to the plunger; the time required to extrude 0.2 mL was recorded to 0.01 s with the stopwatch function of a smartphone (iPhone; Apple Inc., Cupertino, CA, USA), started and stopped by the operator. Measurements were made at an ambient room temperature of 25 °C; the temperature was recorded but not actively controlled, and neither the syringes nor the samples were thermostatted. A 60 s observation limit was set in advance: a fluid that had not extruded 0.2 mL within 60 s was to be recorded as non-extruding. Two fluids reached this limit. Vial B alone was stopped at 60 s and recorded as non-extruding; it is the only fluid for which the pre-set rule was applied as written. One replicate of A + B also passed 60 s without completing; on that occasion the observation was continued rather than stopped, in order to record how long extrusion in fact took, and it completed at 107.97 s. This was a departure from the pre-set rule, decided during the experiment, and is reported here for transparency. That value is a completed extrusion and is treated as one. We distinguish it from the result for vial B alone, which is censored: there we know only that the extrusion time exceeded 60 s, and the fluid is accordingly reported as non-extruding at that load rather than given a number. No numerical extrusion time reported in this paper is a censored value, and no censored value enters any summary statistic. Because the extrusion data are summarized by the median (Section 2.4), the summary reported for A + B is the same whether this replicate is taken at its observed value or treated as censored at 60 s: the median is 39.63 s in either case. Of the five A + B replicates, one exceeded the 60 s limit and four did not; no replicate of any other fluid reached it, and vial B alone failed to extrude on every attempt. This first session was performed on 20 September 2026. Fluids tested were sterile water for injection, 0.9% *w*/*v* sodium chloride injection, A + saline, and A + B. Syringe and needle were replaced when changing fluids, and extruded material was recovered and re-aspirated between replicates of the same fluid.

Replicates of a given fluid were performed sequentially on a single sample. Each replicate required approximately 1–2 min of handling—aspiration, exclusion of air, mounting, loading, timing, and recovery—in addition to the extrusion time itself; thus, a series of five to twelve replicates occupied roughly 10–20 min. With the first measurement begun within 5 min of reconstitution, the five replicates of A + B therefore spanned approximately 5–20 min after mixing; the exact time of each individual measurement was not recorded. All fluids were measured under the same protocol; thus, any systematic drift with measurement order would be expected to affect them equally. We note that replicates of a given fluid are not independent: each series was obtained from a single reconstituted sample by repeated aspiration and extrusion, so elapsed time since reconstitution and cumulative handling of the sample co-vary by construction (Section 4.3). Measurements are reported in the order obtained.

One replicate of A + saline was excluded because of a documented malfunction of the applied weight during that measurement. Vial B alone was tested three times at the 50 g load used for every other fluid and did not complete the 0.2 mL extrusion within 60 s on any of the three attempts.

A second measurement session was performed on 8 October 2026, after the first version of this manuscript had been submitted and reviewed, in order to characterize vial B alone, which the first session had been unable to measure. It used the same syringe and needle types, the same 0.2 mL volume and the same ambient temperature of 25 °C as the first session, and the same lot of vial B as the first session (lot 26B01). The applied load was doubled to 100 g, at which vial B does extrude. Three vials of B were opened in succession and five sequential measurements were made on each, so that the three vials are independent of one another while the five measurements within a vial are not. 0.9% *w*/*v* sodium chloride injection was measured in the same session at the same 100 g load, as a reference fluid of known Newtonian behavior against which the response to the change in load could be judged. Vial B diluted 1:1 with 0.9% *w*/*v* sodium chloride injection was measured at approximately 1 min and again at approximately 15 min after mixing, in three replicates each. All measurements of this session are reported in Section 3.3 and Data S3.

This test yields a relative comparison of extrusion behavior under a geometry relevant to the delivery route. It does not measure viscosity, viscoelasticity, yield stress, or shear-rate dependence, and no rheological parameter is reported. Its limitations are addressed in Section 4.3.

### 2.3. Measurement of Osmolality, Specific Gravity and pH

Osmolality (freezing-point depression), specific gravity, and pH were determined at a clinical laboratory (Fukuyama Clinical Laboratory Center Co., Ltd., Fukuyama, Hiroshima, Japan) as single determinations on each freshly reconstituted preparation and on both reference fluids, so that preparations and diluents were measured in the same analytical system. A + B could not be aspirated into the osmometer, and no value was obtained; the instrument failed to draw the sample rather than returning a value outside its range. No viscosity was measured, and the failure is described here as an inability to aspirate rather than attributed to a measured property.

The osmotic contribution of the declared low-molar-mass components of vial A was calculated from the quantitative composition in Table 1 as *c* = (1/*m*_s_) · Σ_i_ (*w*_i_ · *m*_A_ / *M*_i_), where *w*_i_ is the declared mass fraction of component *i*, *m*_A_ the mass of the powder charge (20 mg), *M*_i_ the molar mass of component *i*, and *m*_s_ the mass of solvent (5.0 g, taking the density of the diluent as 1.00 g/mL). The molar masses used were methionine 149.21, trehalose 342.30 (anhydrous), mannitol 182.17, glutamine 146.14 and glutathione 307.32 g/mol. All five are non-electrolytes and were treated as non-dissociating, and ideal-solution behavior was assumed. The mean molar mass of the osmotically active species in the undisclosed fraction of vial A was obtained by inverting the same relation, *M* = *m*_e_ / (*c*_x_ · *m*_s_), where *m*_e_ is the mass of that fraction (19.86 mg, being 99.300% of the charge) and *c*_x_ the measured osmolality excess. The same relation was applied to the declared composition of vial B, with sodium chloride treated as fully dissociating and each organic solute as non-dissociating. The molar masses used were sodium chloride 58.44, 1,2-hexanediol 118.17, butylene glycol 90.12 g/mol, and the individual molar masses of the 17 declared amino acids. Sodium hyaluronate was entered not as polymer chains, whose contribution by particle count is negligible, but as the sodium counterions of its disaccharide repeat unit (401.3 g/mol), which gives an upper bound on that term because counterion condensation on a polyelectrolyte reduces the osmotically active fraction. An osmotic coefficient of 0.93 was applied to sodium chloride; this was obtained from our own measurements rather than assumed, as the 0.9% *w*/*v* sodium chloride diluent has an ideal osmolality of 308 mOsm/kg and measured 286 mOsm/kg in the same analytical system, a ratio of 0.929. All of these quantities are estimates from declared composition, not measurements; they are reported in Section 3.3 and Section 4.2.

### 2.4. Data Handling

The replicates within each series are not independent (Section 2.2). The n reported for each fluid is therefore the number of repeated measurements made on one reconstituted sample, not a number of independently prepared samples, and it does not function as a sample size. Extrusion data are accordingly summarized descriptively as the median and range, and every individual value is reported in the order obtained (Data S1). Means and standard deviations are given alongside for comparability with other reports, but they estimate neither the precision of the method nor the variability between preparations and should not be read as doing so. Osmolality, specific gravity, and pH were measured once; therefore, no measure of dispersion was available. No inferential statistical testing was performed and no *p*-values are reported, nor would the design support them. The association between the measurement order and extrusion time was examined for each fluid using Spearman’s rank correlation, reported as a descriptive coefficient without significance testing. Tied values were assigned the mean of the ranks they span; two of the series contain a tie. As a second, distribution-free summary of the same question, the mean of the first half of each series was compared with the mean of the second half. No test of statistical equivalence was performed, and comparisons between fluids are described qualitatively rather than as demonstrations of equality.

### 2.5. Use of Generative Artificial Intelligence

Generative artificial intelligence tools were used during the preparation of this manuscript. Claude (Anthropic, San Francisco, CA, USA) and ChatGPT (OpenAI, San Francisco, CA, USA) were used to draft and revise the English text, to check grammar and style, to verify the cited references against their primary sources, and to perform arithmetic on the authors’ own measurement data, including the descriptive statistics in Table 2 and the calculated osmotic contribution of the declared low-molar-mass components reported in Section 4.2. No measurement, result or interpretation was generated by these tools. The authors reviewed and edited all output and take full responsibility for the content of this manuscript.

## 3. Results

### 3.1. Extrusion Behavior

The two reconstituted preparations differed by more than an order of magnitude (Table 2). The median extrusion time of A + saline was 3.05 s (range 2.52–3.58 s), of the same order under the present test conditions as water for injection (3.46 s, 3.08–3.96 s) and 0.9% NaCl (4.01 s, 2.98–5.15 s); no test of statistical equivalence was performed and none is implied. A + B gave a median of 39.63 s (range 20.85–107.97 s), 13 times that of A + saline. Reconstitution of the active powder with saline yielded a fluid whose extrusion behavior was close to that of the reference fluids, whereas reconstitution as directed by the manufacturer did not, at the same active concentration. Because each series was obtained by repeated measurement of one reconstituted sample, these values describe the particular samples measured and not the reproducibility of the preparations.

A + B also could not be aspirated into the freezing-point-depression osmometer (Section 3.4), an instrument of entirely different geometry and driving mechanism. Vial B alone did not complete the 0.2 mL extrusion within 60 s on any of three attempts under the 50 g load applied to every other fluid. We note qualitatively that flow began and then ceased within the observation period rather than never starting; this was not quantified and no time course was recorded. Vial B does extrude when the load is doubled, and its behavior under that condition is reported in Section 3.3. Among the reconstituted preparations and reference fluids, one replicate of A + B was the only measurement to exceed the 60 s observation limit; the remaining four A + B replicates and every replicate of the other three fluids completed within it.

### 3.2. Increase in Extrusion Resistance with Sequential Handling

The dispersion of the A + B values was not random. Measurements taken in sequence on a single reconstituted sample, spanning approximately 5 to 20 min after mixing, were 20.85, 27.28, 39.63, 107.97, and 57.70 s. Extrusion time rose progressively across the first four measurements, a 5.2-fold increase, with the fifth lower than the fourth.

Because every fluid was measured under the same sequential protocol over a comparable interval, the three reference fluids provide a check on drift attributable to the procedure itself—operator fatigue, progressive wear of the syringe, or ambient changes. They show none (Table 3). A + B is the only fluid in which extrusion time depends on the measurement order, which makes simple procedural drift an unlikely explanation.

Therefore, the resistance of A + B to extrusion increased over the course of the series and did so in a manner not seen in the reference fluids or in the same active powder reconstituted with saline. The wide range of the A + B values, and the large standard deviation that would be computed from them, reflect this progression within one sample rather than measurement imprecision or variability between preparations; neither quantity estimates the latter. For the three reference fluids the coefficient of variation ranged from 8% to 15%.

We describe this as an increase in extrusion resistance with sequential handling after reconstitution rather than as time-dependent thickening, because the design does not separate the two. The series was obtained by repeated aspiration and extrusion of a single sample; therefore, the elapsed time, cumulative shear history, any structural rearrangement of the hyaluronate, progressive loss of sample on recovery, and evaporation all advanced together. The reference fluids showed no comparable order-dependent change, making simple apparatus- or operator-related drift less likely; however, they are simple Newtonian fluids and cannot exclude handling effects specific to a polymer-containing preparation. Independent reconstitutions measured at predetermined time points would be required to attribute the increase to elapsed time (Section 4.3).

The lower fifth value is compatible with partial, transient breakdown of the network structure by the repeated aspiration and extrusion inherent to the protocol, but five measurements do not establish this and no mechanism is claimed.

A + B was not measured again in the second session, so this progression has not been tested in the fluid in which it was observed. What the second session shows is that the same pattern does not appear in the other fluids examined. In three independently opened vials of B alone, each measured five times in sequence at 100 g over a comparable interval, Spearman’s coefficient between measurement order and extrusion time was −0.20, +0.05 and −0.10 (Section 3.3, Table 4); none shows the pattern seen in A + B. Vial B diluted 1:1 with saline likewise did not slow between approximately 1 min and approximately 15 min after mixing. The progression reported above therefore stands as a single observation in a single fluid at a single load, absent from the other fluids examined since but untested in the fluid in which it was seen, and it should be read accordingly.

### 3.3. The Base Solution Alone, and the Response to a Doubling of the Applied Load

At 50 g, vial B alone did not complete the 0.2 mL extrusion within 60 s on any of three attempts (Section 3.1). At 100 g it extruded readily. Across three independently opened vials of the same lot, each measured five times in sequence, the median extrusion times were 8.03 s (range 6.64–8.64), 5.98 s (5.61–7.88) and 6.48 s (5.74–6.86), with a median of these three vial medians of 6.48 s (Table 4). Because the vials are independent of one another and the measurements within a vial are not, we take the vial medians rather than the pooled fifteen measurements as the representative figure; the pooled median, 6.64 s, differs little from it. The spread of the three vial medians, 5.98 to 8.03 s, is the only indication this study provides of the variability between separately opened containers.

The reference fluid, which was measured at 100 g in this session and at 50 g in the first, makes these numbers interpretable; the comparison between loads is therefore between sessions, not within one. 0.9% *w*/*v* sodium chloride gave a median of 1.585 s at 100 g against 4.01 s at 50 g, a ratio of 2.53; the median of the six values at 100 g lies between the third and fourth of them, 1.58 and 1.59 s, which is why it carries three decimals. For a Newtonian fluid driven by a dead load the ratio would be 2.0 if the entire load reached the fluid; the excess reflects the part of the load consumed by plunger friction, which is approximately constant and therefore weighs more heavily at the lower load. The two saline medians taken together imply a friction term of roughly 17 g-equivalent. Both sessions used the same syringe and needle types and the same lot of vial B, so this comparison is not confounded by a change in consumables or of material.

Vial B did not respond in the same way. In raw terms, doubling the load took it from failure to complete within 60 s to a median of 6.48 s, a ratio of at least 9.3 against 2.53 for the reference fluid measured in the same way; because the 50 g observation is censored, the true ratio may be larger. Allowing for the plunger friction makes the comparison more exact. Applying the scaling derived from saline to the 100 g median of vial B predicts approximately 17 s at 50 g. What was observed at 50 g was failure to complete within 60 s, which is at least 3.6 times slower than that prediction. With the same apparatus and the same consumables, the reference fluid responded to the doubled load as a Newtonian fluid should and the base solution did not. The difference is consistent with an extrusion behavior that depends on the applied load in a way a Newtonian fluid’s would not. It does not establish non-Newtonian behavior, and we do not claim it does: the two loads were measured in sessions eighteen days apart rather than side by side, the plunger friction was inferred from the reference fluid rather than measured, and the 50 g result for vial B is a censored observation. We derive from this comparison no viscosity, yield stress or flow index, and the test cannot provide one.

Vial B diluted 1:1 with 0.9% *w*/*v* sodium chloride gave a median of 3.46 s (2.59–4.08) at approximately 1 min after mixing and 2.88 s (2.51–2.91) at approximately 15 min, three replicates at each time. It did not slow over this interval.

### 3.4. Osmolality, Specific Gravity and pH

A + saline had an osmolality of 374 mOsm/kg, above the physiological range of approximately 275–295 mOsm/kg and above the 0.9% NaCl used as diluent, which measured 286 mOsm/kg in the same analytical system. The excess is therefore approximately 88 mOsm/kg, established between two measured values rather than against a nominal figure. The declared low-molar-mass excipients of vial A account for 0.142 mOsm/kg of this, calculated as set out in Section 2.3 (see also Section 4.2). No value could be obtained for A + B, which would not enter the instrument. Osmolality is a colligative property and is not by itself a measure of tonicity, which depends on which solutes are membrane-impermeant; no tonicity measurement was made.

The specific gravities were 1.000 for water for injection, 1.006 for 0.9% NaCl, 1.008 for A + saline, and 1.011 for A + B. pH was 5.5 for both reference fluids but 5.0 for both reconstituted preparations, so that dissolution of the powder lowered the pH by the same amount irrespective of which diluent was used; vial A therefore appears to dominate the final pH.

## 4. Discussion

### 4.1. The Supplied Vehicle May Be Poorly Suited to Delivery Through Narrow Conduits

Two findings bear on this. The first is the size of the difference, considered together with what in the declared composition accounts for it; the second, taken up below, is more consequential.

In this single comparison, A + B required about 13 times longer to extrude than A + saline through a 26 G orifice under identical load and at the same active concentration. The same preparation could not be drawn into a freezing-point-depression osmometer. Vial B alone did not pass 0.2 mL through a 26 G orifice within 60 s at 50 g, although it did so in a few seconds when the load was doubled (Section 3.3). Three independent observations, differing in apparatus, geometry, and driving mechanism, pointed in the same direction.

The relation of this test to device-assisted delivery deserves explicit consideration, and the relevant difference is not the one we first supposed. The internal diameter of a 26 G needle is approximately 0.26 mm, and the needles of microneedle radiofrequency devices are of comparable diameter: 0.3 mm in the array characterized by Wootten et al. [17]. The channel that remains after the needle is withdrawn is narrower than the needle that made it, by an amount we cannot state, since the tissue recoils and we did not measure it. The orifice used here is therefore of the same order as the conduit of interest rather than an order of magnitude wider, which is the comparison that matters, and it is not a claim about the patent dimensions of a channel in skin. What differs by orders of magnitude is the driving pressure. The present test applied a 50 g dead load to the plunger of a 1 mL syringe, of the order of 10^4^ Pa before plunger friction is deducted; a preparation presented to micro-channels after treatment is drawn in by capillarity and by whatever light pressure is applied by hand, which for a channel of this radius is of the order of 10^3^ Pa at most. Micro-channel delivery differs from the present test in several further respects: the channels are formed in deformable tissue rather than rigid steel, they close over time, interstitial fluid opposes entry, and the applied volume is distributed across many channels in parallel. It has also been suggested that withdrawal of the needle generates a transient negative pressure that draws fluid into the channel. We raise this because it tells against the comparison we are drawing: if such a transient is substantial, the driving pressure available in vivo would be larger than the estimate above and the argument of this paragraph would be correspondingly weaker. We have not found the effect quantified for devices of this kind, and the standard account of delivery through transiently permeabilized skin is instead one of passive diffusion into channels that remain open for a limited period [7,8,9]. These differences preclude quantitative extrapolation, and we attempt none. We note only that a preparation requiring tens of seconds to pass 0.2 mL through an orifice of this size under a load of 50 g is being asked to do something harder, not easier, when it is presented to a channel of similar caliber with no such load behind it. Whether it enters in useful quantity is an empirical question that the present data cannot answer, and that would need to be addressed directly rather than inferred from these measurements.

Nor should it be assumed that resistance to flow is necessarily unfavorable for delivery, and we state the contrary case because our data do not settle it. A preparation that resists flow also resists being wiped away or running off the surface and may remain in contact with the channel openings for as long as they stay open, which matters if transfer is diffusion-limited rather than flow-limited; a viscous vehicle may also be carried into the tissue on the needle itself rather than entering the channel afterwards and may form a depot instead of being cleared. Viscous vehicles are used deliberately for each of these reasons in other settings. Against this, an entangled polymer network of the kind the declared composition implies would be expected to retard the diffusion of particles in the size range attributed to this class of product, so the same structure that retains the vehicle may also retain its payload. We measured neither delivery nor retention, and the present measurements identify this question rather than answer it.

The declared composition (Table 1) points to sodium hyaluronate as the most plausible contributor to the increased extrusion resistance. At 0.750% *w*/*w* (7.5 mg/mL), it is the only macromolecule declared at a stated quantity in either vial. The other declared solutes in vial B are small molecules present at 0.8% *w*/*w* or less and would not be expected to account for the marked difference in extrusion behavior at the declared concentrations. High-molecular-weight hyaluronan solutions are reported to be shear-thinning and to form entangled networks whose apparent viscosity depends on chain length and concentration, with molecular weight the stronger determinant of entanglement [18]. We did not measure viscosity, shear-rate dependence, or any other rheological parameter, and the molecular weight of the hyaluronate was neither declared nor measured. The ingredient specification gives only “sodium hyaluronate, 0.750% *w*/*w*”, without a molecular weight or a supplier grade, and on enquiry through the distributor we were informed that this information is not disclosed. The declaration therefore gives the quantity of the polymer but not the parameter that principally governs what that quantity does to the solution. Although the molecular weight is not disclosed, the resistance to flow observed at 7.5 mg/mL is more readily reconciled with a high-molecular-weight grade than with a low-molecular-weight one, since resistance to flow at a fixed concentration rises steeply with chain length [18]. We record this as a qualitative expectation consistent with our observations and not as an estimate: the extrusion test yields no rheological parameter, and no molecular weight can be derived from it. This attribution accordingly rests on composition and remains to be confirmed by rheometry of the base solution alone. Notably, the manufacturer’s specification designates the sodium chloride of vial B as a “viscosity increasing agent” (Table 1). Whatever the basis of that particular assignment—hyaluronan is a polyelectrolyte, and the rheology of its solutions is accordingly sensitive to ionic strength [18]—it indicates that the resistance of the vehicle to flow is a deliberate formulation parameter rather than an incidental property.

A second line of evidence, obtained after review, bears directly on this attribution. Doubling the applied load from 50 to 100 g changed the extrusion time of the reference saline by a factor of 2.5, close to the factor of 2 expected of a Newtonian fluid once the constant friction of the plunger is allowed for. The same change in load took vial B alone from failure to complete within 60 s to a median of 6.48 s across three independently opened vials, which at 100 g is at least 3.6 times faster than the saline scaling applied to its own 50 g result predicts (Section 3.3). A Newtonian fluid measured under identical conditions would not behave in this way. The inference rests on the two sessions being comparable, for which the reference fluid measured in both is our only check; we did not measure the plunger friction directly, although the conclusion does not depend on its value, since any friction between zero and twice our estimate still leaves the observed result well outside the Newtonian prediction. The observation is consistent with the shear-thinning behavior reported for entangled hyaluronan solutions [18], and with a flow threshold lying between the stresses applied at the two loads, but the test distinguishes neither and we claim neither. What it does show is that the base solution and a Newtonian reference, measured with the same apparatus and the same consumables, responded very differently to the same change in load. We do not read that as establishing non-Newtonian behavior, for the reasons set out in Section 3.3.

The second finding was that the extrusion resistance of A + B increased more than fivefold over a series spanning the first 20 min after reconstitution, while the same active in saline did not change over a comparable interval (Table 3). This was a single observation, and it was not seen in three independently opened vials of the base solution, nor in the base solution diluted with saline over a comparable interval (Section 3.3); A + B itself was not measured again, so the observation has not been tested in the fluid that produced it. Set beside the first session, the pattern across the four fluids is more informative than the single series alone: of the preparations measured in sequence, the only one in which extrusion time rose was the one containing the contents of both vials. The active powder reconstituted with saline did not rise over a comparable interval, and neither did the base solution with no powder in it, whether undiluted or diluted 1:1 with saline. Whatever produced the rise therefore appears to require both components, not either alone.

Two explanations are consistent with that pattern, and we can exclude neither. The simpler is that dissolution of the lyophilized cake was still proceeding during the series. The first measurement began within 5 min of reconstitution, and a 20 mg cake may dissolve more slowly in a viscous base solution than in saline; if solute was still entering solution as the series progressed, resistance would rise for that reason alone, and would not rise in saline, where dissolution is faster, or in the base solution, where there is nothing to dissolve. The second is an interaction between solutes carried by the active powder and the hyaluronate network of the base solution; divalent cations of the kind present in culture media associate with polyanions, and such association proceeds over minutes rather than instantly. The first of these should be excluded before the second is entertained, and excluding it requires only that the preparation be dissolved to completion before the series begins. We did not do this, and we report the observation with that limitation attached. As set out in Section 3.2, the design does not separate elapsed time from handling history; therefore, the practical implication should be stated conditionally. If the effect reflects change with time after mixing, the fluid presented to the skin would depend on the time elapsed since reconstitution, and a vehicle behaving in this way may compromise the reproducibility of delivery, irrespective of how much or little enters the tissue. If instead it reflects the cumulative shear of repeated handling, the implication differs but is not reassuring, since a delivery procedure also handles the fluid repeatedly. For surface application, either possibility is immaterial, and a film that sets on the skin may even be desirable. For delivery through a device, both are potentially problematic. Distinguishing them is the first experiment we would add.

This matters beyond the specific product, and the simplicity of vial B sharpens rather than weakens the point. The problem is not an elaborate cosmetic emulsion carrying incidental excipients into the dermis; it is the single ingredient that the base solution exists to provide. Sodium hyaluronate is near-ubiquitous in vehicles of this class precisely because it delivers the surface properties that a topical product needs. The two-vial format is standard for lyophilized cosmetic actives, and the supplied base solution is the vehicle the product presents for use. We have not surveyed what practitioners combining such products with energy-based devices in fact do, and the argument here does not depend on the answer. If the supplied base is used as directed, it is used without its extrusion behavior, osmolality or pH having been established; if another diluent is substituted, the substitute is used without those properties having been established either. In neither case are they stated on the product or derivable from its declaration. This base is formulated for a purpose—retention on the skin surface—directly opposed to the requirement of micro-channel delivery. In the single product examined here, that opposition was substantial rather than marginal. Whether it is substantial in this class of vehicle generally is not established by one product and one lot and is the question these observations are intended to raise.

### 4.2. The Osmolality of the Preparation Was Not Predictable from That of the Diluent

Physiological saline was used as a diluent in the expectation that the resulting preparation would have an osmolality near that of extracellular fluid. On the single determination made, A + saline was 374 mOsm/kg at the manufacturer’s own specified reconstitution volume, and therefore at the concentration at which the product is intended to be used. This value was not replicated, and the discussion that follows should be read as an interpretation of one measurement. This was not an artifact of concentrating the preparation. Osmolality is a colligative property that reflects the total number of particles; whether the preparation is hypertonic in the physiological sense depends on which of those particles are membrane-impermeant, which we did not determine.

The quantitative composition allows the source of the excess to be located, and the answer is instructive. The declared low-molar-mass components of vial A—methionine, trehalose, mannitol, glutamine, and glutathione—together amount to 0.70% of a 20 mg charge, or 0.14 mg. Dissolved in 5 mL, their combined osmotic contribution is 0.142 mOsm/kg by the calculation given in Section 2.3: 0.16% of the 88 mOsm/kg by which the measured value exceeds the measured osmolality of the diluent. The declared excipients therefore cannot account for this excess. Nor can the diluent, whose sodium chloride content (0.9% *w*/*v*) is fixed and known.

The remaining 99.3% of the powder was declared under the single INCI designation “Human Renal Stem Cell Conditioned Media Extract”. At 19.9 mg in 5 mL—approximately 4 mg/mL—an osmotic contribution of 88 mOsm/kg corresponds to a mean molar mass of 45.1 g/mol per osmotically active particle (Section 2.3), which is the order expected of inorganic salts rather than of proteins or vesicles. The most parsimonious interpretation is that residual culture-medium salts within the conditioned medium extract carry the osmotic load, the active fraction itself being a minor contributor by particle count. This estimate assumes ideal-solution behavior and complete dissociation and should be regarded as exploratory rather than as a principal result, although the excess it seeks to explain is now bounded by two measurements made in the same system rather than by comparison with a nominal value.

This has a more general consequence than the product examined. A single INCI designation may denote a complex mixture whose composition is undisclosed, and here, that mixture constitutes 99.3% of the reconstituted solid and essentially all of its osmotic activity. The same limitation applies, in a different form, to the one declared component whose identity is unambiguous: the specification gives the quantity of sodium hyaluronate but not its molecular weight, which is what determines its effect on the solution (Section 4.1). In both cases the declaration is complete as a list and uninformative as a prediction. On this evidence the osmolality of the final preparation was not calculable from the ingredient declaration, complete as that declaration appears. It had to be measured. We report the value as found, rather than as a flaw corrected in advance, because this is the more useful point.

Vial B also contains solutes not present in saline—notably 1,2-hexanediol at 0.700% *w*/*w*—so the manufacturer-directed preparation is expected to have a higher osmolality than the saline-reconstituted one, not a lower one. The specification resolves the composition of vial B completely, so the expected value can be calculated by the method of Section 2.3. Sodium chloride contributes approximately 255 mOsm/kg, 1,2-hexanediol 59, the sodium counterions of the hyaluronate at most 19, butylene glycol 17 and the 17 amino acids 5, giving approximately 354 mOsm/kg for vial B alone. Adding the 88 mOsm/kg that vial A was measured to contribute gives an expected osmolality of approximately 440 mOsm/kg for the manufacturer-directed preparation, against the 374 mOsm/kg measured for the saline-reconstituted one. This figure rests on the assumption that the contribution of vial A is additive across diluents, which we did not test, and on ideal-solution behavior; it is an estimate offered because no measurement could be obtained, and it should not be read as one. The direction it indicates is the one the composition already implied: substituting saline for vial B lowers the osmolality of the preparation rather than raising it, by approximately 66 mOsm/kg on this calculation. The substitution therefore changes both properties, but not in the same proportion: it alters the extrusion behavior by more than an order of magnitude and the osmolality by under a fifth. It is worth noting why the osmolality changes at all, since the sodium chloride concentrations of the two diluents differ by only 0.1 percentage point: the difference is carried by the other solutes of vial B, principally 1,2-hexanediol, with smaller contributions from butylene glycol, the counterions of the hyaluronate and the amino acids. Neither diluent brings the preparation into the physiological range.

Bringing the final preparation closer to the osmolality of the extracellular fluid would require a diluent with a lower osmolality than physiological saline. Its composition needs to be established experimentally, along with confirmation of formulation stability and biological compatibility.

The pH findings follow the same pattern. At pH 5.0, both preparations were appreciably more acidic than the dermal interstitium. Both diluents measured pH 5.5; therefore, dissolution of vial A lowered the pH by the same increment in each case, and the two preparations converged on the same value despite differing in vehicle. Vial A therefore appears to dominate the final pH across the two diluents examined. We do not extend this to diluents we did not test: an unbuffered diluent would not be expected to hold the pH, but a buffered one might, and that is an experiment rather than an inference. A pH near 5 is appropriate for a product intended for the skin surface, where the acid mantle lies in the same range, and may additionally reflect the stabilization of the lyophilized formulation; it is not obviously appropriate for a fluid placed in the reticular dermis. Whether a buffered reconstitution medium bringing the final pH nearer the physiological range would alter either the activity or tolerability has not been examined and merits study.

### 4.3. Limitations

This is a preliminary study, and the weight that can be placed on it is correspondingly limited. It examines a single production lot of a single commercial product, compares two preparations of it, and rests on repeated measurements of one reconstituted sample of each fluid together with single determinations of osmolality, specific gravity and pH. It is intended to establish that differences large enough to be worth investigating exist, and to identify which measurements are needed; it is not a characterization of the product, of the manufacturer’s base solution as a class of vehicle, or of reconstitution vehicles in general. Every statement below about what the findings may imply is conditional on confirmation by the experiments set out at the end of this section.

Replicates within each series were not independent, and this was the principal limitation of the study. The second measurement session goes part of the way toward remedying it for one fluid: the three vials of base solution were opened independently of one another, so the three vial medians are independent observations, and the agreement among them is the only estimate of between-container variability this study contains. The five measurements within each vial remain non-independent, and no independent replication exists for either reconstituted preparation, which are the fluids the paper is about. The second session did not re-measure A + B: it was undertaken to characterize the base solution alone, which the first session had been unable to measure, and independent reconstitutions of A + B at predetermined time points were not possible within the revision period. The progressive increase reported in Section 3.2 has therefore not been tested again in the fluid in which it was seen, and the statement there that the pattern is absent from the other fluids examined is not a failed replication of the A + B series. Each series was obtained from a single reconstituted sample by repeated aspiration and extrusion. Elapsed time since reconstitution, cumulative shear history, any structural rearrangement of the hyaluronate, progressive loss of sample on recovery, and evaporation therefore advanced together and could not be separated. The reference fluids (Table 3) showed no comparable order-dependent change, making simple apparatus- or operator-related drift less likely; however, they are simple Newtonian fluids and cannot exclude handling effects specific to a polymer-containing preparation. The increase in extrusion resistance reported in Section 3.2 was accordingly described as occurring with sequential handling after reconstitution, and not as a measured time course. Independent reconstitutions measured at predetermined time points—the experiment we regard as most urgently needed—would be required to establish the kinetics of change with time.

The extrusion test is a relative method. It uses a fixed orifice and a fixed load; therefore, the samples flow at a single, uncharacterized shear condition; it yields no viscosity, viscoelastic modulus, or yield stress, and its values cannot be compared with the rheological literature. It does not characterize the shear-rate dependence that a preparation containing high-molecular-weight polymers would be expected to display. Rotational rheometry of both preparations—flow curves across the relevant shear-rate range, together with a time sweep and a determination of yield stress, the last because the base solution began to flow and then ceased within the observation period (Section 3.1)—is the necessary next step and would allow the argument in Section 4.1 to be stated quantitatively. No rheological parameters are claimed in this report. The pre-set 60 s observation limit was not applied uniformly: one replicate of A + B was allowed to run past it and was timed to completion (Section 2.2). The median summary is unaffected by whether that replicate is treated as a completion or as censored at 60 s, although the mean and standard deviation are not, which is a further reason for not relying on them. The two measurement sessions were separated by eighteen days and conducted at different applied loads, so the comparison between them in Section 3.3 rests on the constancy of the apparatus and of the operator’s technique over that interval. The syringe and needle types and the lot of vial B were the same in both, and the reference fluid was measured in both, which is the strongest check available to us but is not a calibration. Times were read to 0.01 s but started and stopped by hand, so the effective resolution is set by the operator’s reaction time rather than by the display; for the reference fluids, whose extrusion times are of the order of a few seconds, this is a material part of the observed scatter. Syringe plunger friction contributes to the measured times; the coefficient of variation of 8% for water for injection indicates that this contribution was reasonably consistent but does not eliminate it.

The time of each individual measurement was not recorded; therefore, the A + B series is bounded by an estimated interval of approximately 5–20 min after reconstitution rather than anchored to defined time points.

Osmolality, specific gravity, and pH were single determinations performed in a clinical laboratory on a system calibrated for biological fluids; replicate measurements on independently reconstituted samples on a system calibrated for the sample type would be required to establish precision. Preparations and reference fluids were measured in the same analytical system, so the excess attributed to the powder rests on two measured values rather than on comparison with a nominal figure. No osmolality value was obtained for A + B.

Several constraints follow from working with declared rather than measured composition. The quantitative composition used here is taken from the manufacturer’s ingredient specifications and has not been independently verified by the authors. The molecular weight of the sodium hyaluronate, which is the stronger determinant of entanglement and hence of solution rheology [18], is neither declared nor measured, and was not disclosed on enquiry; therefore, the attribution in Section 4.1 rests on composition alone. Measuring it, or obtaining it from the manufacturer, would be required to confirm that attribution. No measured osmolality could be obtained for the manufacturer-directed preparation, and the figure of approximately 440 mOsm/kg given for it in Section 4.2 is calculated from declared composition rather than measured. It assumes ideal-solution behavior and that the contribution of vial A is additive across diluents, neither of which we tested, and it is reported as an estimate in place of a measurement rather than alongside one. The estimate of the mean molar mass of the osmotically active species in the conditioned medium extract assumes ideal-solution behavior and complete dissociation and is exploratory rather than a principal result.

What was measured should not be read as more than it is. Osmolality is a colligative property; tonicity, which depends on solute permeability across cell membranes, was not measured and cannot be inferred from it. The presence, quantity, and integrity of Klotho protein and extracellular vesicles were likewise not verified: no immunoassay, particle tracking, or tetraspanin analysis was performed, and the preparation has not been characterized in accordance with MISEV2023 [2]. References to these components in this report, and to Klotho and extracellular vesicles in the Introduction, reflect the manufacturer’s product description and the published literature only. Nothing in this study bears on whether these components are present in the product, whether they survive reconstitution, or whether they reach or act within the skin by any route. Nor does this study examine delivery through micro-channels, which was not performed; the geometric comparison in Section 4.1 is an argument about orifice dimensions and not a delivery experiment. No clinical significance of any kind is claimed or implied.

Finally, measurements were made on a single production lot of each product; therefore, lot-to-lot variability was not assessed. This study reports the properties of the fluid presented to the skin; it makes no measurement of what crosses the barrier, and no claim regarding delivery, retention, or biological activity.

## 5. Conclusions

In this preliminary comparison, the cosmetic base solution supplied with a lyophilized stem cell conditioned medium extract produced a preparation whose median time to pass a 0.26 mm orifice was about 13 times that of the same active, at the same concentration, reconstituted with saline. The same preparation would not enter a second instrument of different geometry, and the base solution alone did not pass the orifice within 60 s at 50 g. When the load was doubled to 100 g the base solution did extrude, with vial medians of 8.03, 5.98 and 6.48 s across three independently opened vials; the reference saline responded to that doubling by a factor of 2.5, close to the Newtonian expectation, whereas the base solution was at least 3.6 times slower than the same scaling predicts. We read this as suggesting that its flow depends on the applied load in a way a simple viscous liquid’s does not, while noting that the two loads were measured in separate sessions and that we did not determine any rheological parameter. Across the sequence of measurements made on one sample of that preparation, spanning roughly the first 20 min after mixing, extrusion resistance rose more than fivefold. Because those measurements were made on a single sample by repeated handling, elapsed time and handling history are not separated, and distinguishing them is the first thing a further study should resolve.

Reconstitution with physiological saline yielded a fluid whose extrusion behavior approached that of water but not, on the single determination made, one with physiological osmolality: 374 mOsm/kg at the manufacturer’s own use concentration, against 286 mOsm/kg for the diluent measured in the same system. The declared excipients of the powder account for 0.16% of that 88 mOsm/kg excess; the remainder is most plausibly attributable to the conditioned medium extract itself, a mixture constituting 99.3% of the powder and declared under a single INCI name without further disclosure. Both diluents had a pH of 5.5 and both reconstituted preparations a pH of 5.0, so the choice of diluent did not alter the final pH of the preparation.

These are observations on one lot of one product, obtained without independently prepared replicates, and they establish no general property of reconstitution vehicles. What they do suggest is a question worth putting to such products directly: for this preparation, neither the extrusion behavior nor the osmolality could have been anticipated from the ingredient declaration—the former because the molecular weight of the declared polymer is not stated and was not disclosed on enquiry, the latter because the dominant constituent is an undisclosed mixture recorded under a single INCI name. Where a product of this class is being considered for a route other than surface application, measuring these properties on the final reconstituted fluid appears more reliable than inferring them from the label.

These measurements should not be read as recommending reconstitution with saline. Substituting saline addressed the extrusion behavior but left the preparation at 374 mOsm/kg and at pH 5.0, and neither preparation was assessed for cell compatibility, tolerability, or retention of the declared active components; no delivery of any kind was measured, for either preparation. What these measurements do support is narrower and, we think, more useful: that the extrusion behavior differed by more than an order of magnitude between the two vehicles while the pH did not differ at all, that the osmolality of the manufacturer-directed preparation could not be measured, that none of the three was predictable from the ingredient declaration, and that they should be determined on the final preparation before a vehicle is chosen for any route other than surface application.

Independent reconstitutions measured at predetermined time points, replicate physicochemical determinations on a system calibrated for the sample type, rotational rheometry of both preparations including a determination of yield stress (Section 4.3), and analysis of more than one production lot are the necessary next steps, and are required before any of the above can be stated as more than a preliminary observation.

## Figures and Tables

**Table 1:** Declared quantitative composition of the two vials, from the manufacturer’s ingredient specifications (Elastem Co., Ltd.: document no. 240516-00 of 16 May 2024 for vial A; document no. 231215-03 of 19 December 2023 for vial B). The last three entries for vial B are declared in the specification as a premix totaling 0.500% *w*/*w*; the specification resolves the individual quantities within it, and they are reproduced here. The 17 free amino acids are listed individually in the specification and are summed here; glycine (0.01225%) and serine (0.00680%) are the largest.

Vial	Component	% *w*/*w*	Declared Function
A (20 mg powder)	Human renal stem cell conditioned media extract	99.300	Skin conditioning
	Methionine	0.220	Skin conditioning
	Trehalose	0.200	Skin conditioning
	Mannitol	0.100	Skin conditioning
	Glutamine	0.100	Skin conditioning
	Glutathione	0.080	Reducing agent
B (5 mL solution)	Water	97.249	Solvent
	Sodium chloride	0.800	Viscosity increasing agent
	Sodium hyaluronate	0.750	Skin conditioning
	1,2-Hexanediol	0.700	Solvent
	Copper tripeptide-1	0.001	Skin conditioning
	Premix: water	0.300	Solvent
	Premix: butylene glycol	0.150	Skin conditioning
	Premix: 17 free amino acids (sum)	0.050	Skin conditioning

**Table 2:** Physicochemical properties of the two reconstituted preparations and the reference fluids. n denotes the number of repeated measurements made on a single reconstituted sample of each fluid, not the number of independently prepared samples; the replicates are therefore not independent (Section 2.2 and Section 2.4). Extrusion conditions: 26 G orifice, 50 g load, 0.2 mL, 25 °C. Osmolality, specific gravity and pH are single determinations. n.m., not measurable; SD, standard deviation. ᵃ One replicate excluded owing to a documented malfunction of the applied weight. ᵇ Individual values in measurement order: 20.85, 27.28, 39.63, 107.97, 57.70 s; one of the five exceeded the 60 s observation limit (Section 2.2). ᶜ A + B could not be aspirated into the osmometer.

Property	Water for Injection	0.9% NaCl	A + Saline	A + B
Extrusion time for 0.2 mL, median (range), s	3.46 (3.08–3.96)	4.01 (2.98–5.15)	3.05 (2.52–3.58) ᵃ	39.63 (20.85–107.97) ᵇ
Extrusion time, mean ± SD, s	3.50 ± 0.28	4.09 ± 0.61	3.06 ± 0.34	50.69 ± 34.97
Repeated measurements on one sample (n)	10	12	9	5
Ratio of medians to A + saline	1.13	1.31	1.00	13.0
Osmolality (mOsm/kg)	0	286	374	n.m. ᶜ
Specific gravity	1.000	1.006	1.008	1.011
pH	5.5	5.5	5.0	5.0

**Table 3:** Dependence of extrusion time on measurement order within each series. All replicates were obtained from a single reconstituted sample per fluid and are not independent; see Section 2.4 and Section 3.2. ρ is Spearman’s rank correlation between measurement order and extrusion time; the final column compares the mean of the first half of each series with that of the second half.

Fluid	n	ρ	First Half → Second Half
A + B	5	+0.90	24.07 → 68.43 s (+184%)
A + saline	9	+0.22	3.02 → 3.10 s (+2.6%)
Water for injection	10	−0.22	3.53 → 3.48 s (−1.6%)
0.9% NaCl	12	+0.01	4.03 → 4.16 s (+3.3%)

**Table 4:** Extrusion behavior at a doubled load of 100 g, measured on 8 October 2026 with the same syringe and needle types as the first session and the same lot of vial B. Three vials of B were opened in succession and measured five times each: the three vials are independent of one another, the five measurements within a vial are not. 0.9% NaCl was measured in the same session as a reference. ρ is Spearman’s rank correlation between measurement order and extrusion time within each series; it is reported as a descriptive coefficient without significance testing. Conditions otherwise as in Table 2.

Fluid	n	Median (Range), s	ρ
0.9% NaCl	6	1.585 (1.29–2.63)	−0.49
Vial B alone, vial 1	5	8.03 (6.64–8.64)	−0.20
Vial B alone, vial 2	5	5.98 (5.61–7.88)	+0.05
Vial B alone, vial 3	5	6.48 (5.74–6.86)	−0.10
Vial B + saline 1:1, ~1 min after mixing	3	3.46 (2.59–4.08)	—
Vial B + saline 1:1, ~15 min after mixing	3	2.88 (2.51–2.91)	—

## Data Availability

The original contributions presented in this study are included in the article and in the Supplementary Material. Further inquiries can be directed to the corresponding author.

## Supplementary Materials

The following supporting information can be downloaded at: https://www.mdpi.com/article/10.3390/molecules31214033/s1, Data S1: Extrusion test—every individual measurement, in the order obtained; Data S2: Osmolality, specific gravity and pH—single determinations; Data S3: Second session (8 October 2026)—base solution alone at a doubled load.
