# Supplementary material for: Extrusion Behavior, Osmolality and pH of a Reconstituted Conditioned-Medium Cosmetic Product: A Pilot Physicochemical Comparison of Two Reconstitution Vehicles
Source: Molecules. 2026 Nov 9;31(21):4033. doi: 10.3390/molecules31214033 (PMC13764759; doi:10.3390/molecules31214033)
Supplement: Supplementary file 1 [file molecules-31-04033-s001.zip › molecules-4608288-supplementary.pdf]

Supplementary Data S1–S3 — Complete Primary Measurements

Extrusion Behavior, Osmolality and pH of a Reconstituted Conditioned-Medium Cosmetic Product: A Pilot Physicochemical Comparison of Two Reconstitution Vehicles | *Nemoto M, Yamada H*

Data S1. Extrusion test — every individual measurement, in the order obtained

Conditions. 1 mL syringe with a 26 G needle, fixed vertically; constant 50 g load on the plunger; time to extrude 0.2 mL recorded to 0.01 s; room temperature 25 °C. A 60 s observation limit was set in advance, beyond which a measurement was to be recorded as non-extruding. Two fluids reached it. Vial B alone was stopped at 60 s and recorded as non-extruding. One replicate of A + B also passed 60 s without completing; that observation was continued rather than stopped, in order to record how long extrusion in fact took, and completed at 107.97 s. This was a departure from the pre-set rule, decided during the experiment. That value is a completed extrusion and is treated as one. The result for vial B alone at 50 g is different: there the extrusion did not complete, so the time is right-censored at 60 s and no number is given for it. No numerical extrusion time in these tables is censored. Because the summary below uses the median, the summary for A + B is the same whether that replicate is taken at 107.97 s or treated as censored at 60 s: the median is 39.63 s either way. Syringe and needle were replaced when changing fluids, and extruded material was recovered and re-aspirated between replicates of the same fluid. The first measurement of each series began within 5 min of reconstitution; each replicate required approximately 1–2 min of handling in addition to the extrusion time, so the five replicates of A + B span approximately 5–20 min after mixing. The exact time of each individual measurement was not recorded.

Replicates within a series are not independent. Each series was obtained from a single reconstituted sample by repeated aspiration and extrusion, so elapsed time since reconstitution and cumulative handling of the sample co-vary by construction. The n of each series is a number of repeated measurements on one sample, not a number of independently prepared samples. See Sections 2.4, 3.2 and 4.3 of the manuscript.

Replicate order	1	2	3	4	5	6	7	8	9	10	11	12
Water for injection	3.71	3.08	3.41	3.66	3.80	3.96	3.36	3.35	3.50	3.21	—	—
0.9% NaCl injection	4.41	2.98	3.60	3.96	5.15	4.06	4.48	4.98	3.78	4.31	3.63	3.78
A + saline	2.52	3.25	**5.55 ***	3.58	2.73	2.91	2.95	3.45	3.13	3.05	—	—
A + B	20.85	27.28	39.63	107.97	57.70	—	—	—	—	—	—	—

All values in seconds. ***** A + saline, replicate 3 (5.55 s) was excluded from the summary statistics because a malfunction of the applied weight was documented during that measurement; the value is reported here for completeness. **Vial B alone** was tested at the 50 g load used for every other fluid and, after it failed to extrude, at a doubled load of 100 g. At 50 g it did not complete the 0.2 mL extrusion within 60 s on any of three attempts; at 100 g all fifteen measurements completed (Data S3). Flow began and then ceased within the observation period rather than never starting; this was not quantified and no time course was recorded. The 100 g attempts are not comparable with the values tabulated above, all of which were obtained at 50 g.

Descriptive summary of these values (Table 2 of the manuscript)

Fluid	n	Median (range), s	Mean ± SD, s	CV (%)	Ratio of medians to A + saline
Water for injection	10	3.46 (3.08–3.96)	3.50 ± 0.28	7.9	1.13
0.9% NaCl injection	12	4.01 (2.98–5.15)	4.09 ± 0.61	14.9	1.31
A + saline	9	3.05 (2.52–3.58)	3.06 ± 0.34	10.9	1.00
A + B	5	39.63 (20.85–107.97)	50.69 ± 34.97	69.0	13.0

Computed from the values above, excluding the replicate marked *. n is the number of repeated measurements on a single reconstituted sample. Mean, SD and CV are given for comparability only; they estimate neither the precision of the method nor the variability between independently prepared samples. Table 3 of the manuscript reports Spearman's rank correlation between replicate order and extrusion time for each fluid, calculated from these same values.

Data S2. Osmolality, specific gravity and pH — single determinations

Conditions. Determined at Fukuyama Clinical Laboratory Center Co., Ltd. (Fukuyama, Hiroshima, Japan) on freshly reconstituted preparations and on both reference fluids, measured in the same analytical system. Osmolality by freezing-point depression. No replicate measurements were made, so no measure of dispersion is available for these parameters (Section 4.3 of the manuscript).

Fluid	Osmolality (mOsm/kg)	Specific gravity	pH
Water for injection	0	1.000	5.5
0.9% NaCl injection	286	1.006	5.5
A + saline	374	1.008	5.0
A + B	not measurable †	1.011	5.0

† The instrument failed to draw the sample rather than returning a value outside its range. No viscosity was measured, and the failure is reported here as an inability to aspirate rather than attributed to a measured property (Section 2.3 of the manuscript).

Data S3. Second session (8 October 2026) — base solution alone at a doubled load

Conditions. Same syringe and needle types as Data S1 (Terumo 1 mL tuberculin syringe; NIPRO 26 G × 13 mm needle, 0.45 mm outer diameter), same 0.2 mL volume, same ambient temperature of 25 °C, and the same production lot of vial B (lot 26B01). Applied load doubled to 100 g, at which vial B does extrude. Three vials of B were opened in succession and measured five times each: **the three vials are independent of one another; the five measurements within a vial are not.** 0.9% w/v sodium chloride was measured in the same session at the same load, as a reference fluid of known Newtonian behaviour. Values in seconds, in the order obtained.

Fluid	1	2	3	4	5	6	Median	Range
0.9% NaCl (100 g)	1.88	1.58	2.63	1.29	1.59	1.36	1.585	1.29–2.63
Vial B alone — vial 1	8.64	7.46	6.64	8.24	8.03	—	8.03	6.64–8.64
Vial B alone — vial 2	5.61	7.88	7.01	5.98	5.98	—	5.98	5.61–7.88
Vial B alone — vial 3	6.28	6.54	6.86	5.74	6.48	—	6.48	5.74–6.86
Vial B + saline 1:1, ~1 min	3.46	4.08	2.59	—	—	—	3.46	2.59–4.08
Vial B + saline 1:1, ~15 min	2.88	2.51	2.91	—	—	—	2.88	2.51–2.91

A practice run preceding the session gave 0.58 s for air and 1.33 s for 0.9% NaCl; these are not included above. At 50 g in the first session, vial B alone was tested three times and did not complete the extrusion within 60 s on any attempt. Spearman's rank correlation between measurement order and extrusion time was –0.49 for the saline series and –0.20, +0.05 and –0.10 for the three vial B series; none shows the progressive increase reported for A + B in the first session.

Preparations

A + saline — vial A (20 mg lyophilized powder) reconstituted with 5.0 mL of sterile 0.9% w/v sodium chloride injection. **A + B** — vial A reconstituted with the full 5.0 mL content of vial B (the manufacturer's Care Solution), as directed by the manufacturer. The same volume of diluent, 5.0 mL, was added to the powder in both preparations; neither was afterwards made up to a fixed final volume, and neither volume was verified gravimetrically. The concentration of the active was therefore identical in the two preparations, which differed only in the identity of the diluent. **These three tables contain every measurement used in the analyses reported in the paper, from both sessions.** The only other readings taken were the practice run noted under Data S3.
